# Cross-Sectional Associations Between Skeletal Muscle Measurements, Myostatin, and MicroRNA-133a in Heart Failure Patients Undergoing Cardiac Rehabilitation

**DOI:** 10.3390/biomedicines14061243

**Published:** 2026-05-29

**Authors:** Kevin Triangto, Bambang B. Siswanto, Tresia F. U. Tambunan, Teuku Heriansyah, Alida R. Harahap, Aria Kekalih, Hajime Katsukawa, Anwar Santoso, Basuni Radi

**Affiliations:** 1 Eka Hospital MT Haryono, Jakarta 12830, Indonesia; 2 National Cardiovascular Center Harapan Kita, Jakarta 11420, Indonesia; bambbs@gmail.com (B.B.S.); anwarsantoso@inaheart.org (A.S.); basuniradi@gmail.com (B.R.); 3 Doctoral Program in Medical Sciences, Faculty of Medicine, Universitas Indonesia, Jakarta 10430, Indonesia; fransiska_ut@yahoo.com.au (T.F.U.T.); alida_r_harahap@yahoo.com (A.R.H.); 4 Department of Cardiology and Vascular Medicine, Faculty of Medicine, Universitas Indonesia, Jakarta 10430, Indonesia; 5 Department of Physical Medicine and Rehabilitation, Faculty of Medicine, Universitas Indonesia, Jakarta 10430, Indonesia; 6 Department of Cardiology and Vascular Medicine, Faculty of Medicine, Universitas Syiah Kuala, Banda Aceh 23111, Indonesia; teuku_hery@usk.ac.id; 7 Department of Community Medicine, Faculty of Medicine, Universitas Indonesia, Jakarta 10430, Indonesia; aria.kekalih@gmail.com; 8 Department of Scientific Research, Japanese Society for Early Mobilization, Tokyo 102-0073, Japan; winegood21@gmail.com

**Keywords:** 6-Minute Walk Test, myostatin, miRNA, heart failure, cardiac rehabilitation

## Abstract

**Background:** Skeletal myopathy is a common complication of heart failure (HF), contributing to exercise intolerance and impaired physical function. This study explores the relationship between practical skeletal muscle measurements and key biomarkers in HF patients undergoing cardiac rehabilitation. **Methods:** Sixty-nine stable chronic HF patients participated in a 3-month phase II cardiac rehabilitation program. Physical examinations, including the 6-Minute Walk Test (6MWT), chest expansion, inspiratory diaphragm thickness, and handgrip strength, were conducted. Blood samples were analyzed for myostatin and miRNA-133a. Data were analyzed using paired *t*-tests, Wilcoxon tests, Chi-square/Fisher’s exact tests, and correlation analyses. **Results:** Significant improvements were observed in 6MWT distance, chest expansion, and inspiratory diaphragm thickness following rehabilitation (*p* < 0.001). Handgrip strength also significantly improved post-rehabilitation. Myostatin and miRNA-133a levels did not change significantly post-rehabilitation. However, exploratory cross-sectional analysis revealed trends suggesting that lower myostatin levels correlated with better endurance (*p* = 0.036), while higher myostatin levels were also observed in patients with better 6MWT performance (*p* = 0.014). Higher miRNA-133a levels were potentially associated with better overall fitness, including endurance and respiratory function (*p* < 0.05). **Conclusions:** Readily performed physical assessments can serve as clinical indicators of the systemic impact of HF on skeletal muscle. The study highlights the importance of evaluating extracardiac function in HF patients, demonstrating potential exploratory associations between physical function and key biomarkers.

## 1. Introduction

Heart failure (HF) is a major public health concern associated with high rates of morbidity and mortality globally [1]. While primarily a disorder of the heart, HF is increasingly recognized as a systemic disease affecting multiple organs, particularly skeletal muscle. Skeletal myopathy, characterized by reduced muscle mass, strength, and altered muscle fiber characteristics, is a common complication of chronic HF (CHF) [2,3]. This myopathy significantly contributes to exercise intolerance, impaired physical function, and reduced quality of life in HF patients, independent of cardiac function [4,5].

Over the years, cardiac rehabilitation (CR) has played such a pivotal role in HF that recently it has been deemed as the fifth pillar of HF management. Because skeletal myopathy is a key driver of exercise intolerance, structured, exercise-based CR programs are essential therapeutic interventions designed to improve physical capacity and mitigate these systemic effects. By engaging in tailored exercise protocols, HF patients can experience significant improvements in functional capacity, making CR critical to addressing the broader systemic impact of the disease.

Understanding the mechanisms underlying skeletal myopathy in HF is essential for developing effective therapeutic interventions, particularly exercise-based CR [3,6]. Myokines, proteins secreted by muscle fibers in response to contraction, have emerged as potential players in the pathophysiology of skeletal myopathy and adverse cardiac remodeling in HF [7,8,9]. Myostatin, a member of the transforming growth factor-beta (TGF-β) superfamily, is a well-established negative regulator of skeletal muscle growth [9,10]. Elevated myostatin levels have been observed in the serum and skeletal muscle of HF patients and are associated with skeletal muscle wasting [8,9]. MicroRNAs (miRNAs) are small non-coding RNA molecules that regulate gene expression and are involved in various cardiovascular processes, including cardiac hypertrophy, fibrosis, and apoptosis [11,12]. MiRNA-133a is a muscle-specific miRNA known to play roles in muscle proliferation and differentiation, and its dysregulation has been linked to cardiac hypertrophy and inflammation [13,14]. Circulating levels of miRNA-133a have been investigated as potential biomarkers in HF [11,14].

While biomarkers like myostatin and miRNA-133a offer insights into the molecular mechanisms of skeletal myopathy, their measurement typically requires blood sampling and laboratory analysis [9,14,15]. More practical, easily obtainable measures of skeletal muscle function and size could be valuable tools for routine clinical assessment and monitoring in HF patients undergoing CR [16,17]. Handgrip strength is a widely accepted measure of overall muscle strength and has been shown to predict outcomes and frailty in HF [16,18,19]. Muscle thickness, assessable via ultrasonography, provides a direct measure of muscle size [16,20]. However, the direct correlation of these practical physical measurements with key myokines and miRNAs in the context of cardiac rehabilitation for heart failure patients is not yet fully established. Establishing the relationship between these practical physical assessments and key biomarkers like myostatin and miRNA-133a could support the use of physical measurements as accessible proxy indicators of underlying myopathy and its associated biomarker profile.

This study aims to illustrate the relationship between practical skeletal muscle measurements and the biomarkers myostatin and miRNA-133a in HF patients undergoing CR. The central hypothesis is that easily performed physical examinations and measurements can provide valuable information reflective of the status of these important extracardiac biomarkers, offering a more practical approach to assessing skeletal myopathy in the HF population. Furthermore, while a single-group design without a control group limits definitive causal interpretation regarding the rehabilitation itself, this prospective approach is justified to pragmatically explore baseline physical capabilities and short-term functional improvements associated with biomarker profiles.

## 2. Materials and Methods

### 2.1. Setup

This is a prospective single-group etiology study examining the relationship between biomarker changes and extracardiac parameters before and after phase II CR over a three-month duration. Data collection used consecutive sampling from CHF patients participating in phase II CR. The study was conducted at the National Cardiovascular Center Harapan Kita (PJNHK) in Jakarta, Indonesia, from July 2023 to October 2024.

### 2.2. Patient Selection

This study recruited 69 stable CHF patients, aged 18–65 years, diagnosed with CHF primarily due to coronary artery disease or cardiomyopathy, all with reduced ejection fraction below 40% as measured by echocardiography. Prior to enrolment, all patients were receiving optimal and similar standard medical therapy for heart failure. Additionally, the duration since each patient’s initial HF diagnosis was recorded to account for any prior exposure to other cardiac rehabilitation programs. The criteria for patient selection were as follows:

Inclusion Criteria:

1. Stable hemodynamic conditions for at least one week post-treatment;
2. Ability to walk a minimum of 100 m in the 6-Minute Walk Test (6MWT) [21];
3. Having exceeded the myocardial stunning period (3 months post-percutaneous coronary intervention or 6 months post-coronary artery bypass surgery) [22].

Exclusion Criteria:

1. Physical limitations preventing protocol compliance;
2. CHF due to severe valvular problems;
3. Significant extremity pain (with pain score ≥4);
4. Communication disorders;
5. Severe obstructive respiratory disease;
6. Neuromuscular disorders or musculoskeletal problems that interfered with ambulation.

The sample size of 69 patients was pragmatically determined due to funding limitations for biomarker analysis. While this sample size allowed for the detection of significant changes in key physical parameters and cross-sectional correlations, it may have limited the power to detect subtle longitudinal changes in biomarkers or to perform extensive subgroup analyses. A formal power calculation for all outcomes was not performed, which is a limitation to consider.

### 2.3. Data Collection

Baseline data were collected through history taking, physical examination, supporting examinations, and medical record review. Physical examinations included comprehensive assessments such as the 6MWT, five times sit-to-stand test, chest expansion measurement, handgrip strength testing using a dynamometer, and diaphragm and anterior forearm muscle thickness evaluation via ultrasonography (USG) [16,17,23]. These specific clinical examinations and ultrasound assessments formed the core of the phase II evaluation to precisely quantify both baseline status and post-rehabilitation progress. Chest expansion was measured at the third intercostal space (superior) and the xiphoid process/10th thoracic vertebrae (inferior) during maximum inspiration and expiration by trained personnel, ensuring standardized patient positioning [24]. Diaphragm thickness was assessed during inspiration and expiration using USG [5]. Anterior forearm muscle thickness was measured via USG on both the dominant and non-dominant sides [16]. All USG measurements were performed by a single radiologist with over 10 years of experience and a subspecialty in musculoskeletal imaging. Utilizing a single expert operator eliminated the risk of interobserver variability. While specific intra-observer variability was not quantified for this specific cohort, previous studies have established that B-mode ultrasound is a highly reliable and reproducible tool for assessing forearm muscle thickness (with intra-class correlation coefficients >0.92) when performed by experienced practitioners [20,25].

Handgrip strength was measured using a Camry electronic hand dynamometer as published previously, taking the maximum value from three attempts on each hand. To ensure the reliability of these clinical parameters, intra- and interobserver variability were evaluated, with the variability for ultrasound measurements of muscle thickness maintained strictly below 5%. These physical measurements served as practical indicators of muscle strength, endurance, and overall functional capacity relevant to skeletal myopathy, rather than a formal diagnosis of sarcopenia based on comprehensive criteria [16,26].

Regarding potential confounders, specific data on estimated Glomerular Filtration Rate (eGFR), detailed dietary effects, and precise dosages of all optimum medical therapies were not systematically collected as part of this study design. While patients were stable and on standard HF treatment, these factors could potentially influence biomarker levels and physical outcomes and represent areas for consideration in future research.

### 2.4. Laboratory Examination (Biomarkers)

Laboratory tests were performed by trained analysts using peripheral blood samples collected serially at the initial and final assessment sessions of the rehabilitation program. The analyzed biomarkers were:

- Myostatin: Plasma EDTA samples were obtained from blood collection, followed by a centrifugation for 15 min at 1000× *g* within 30 min of collection. Samples were aliquoted and stored at −80 °C until analysis. The ELISA kit was GDF8 (#DGDF80; R&D Systems Europe, Abingdon, UK), used according to the manufacturer’s instructions. The optical density was measured using a microplate reader (Multiskan Go, ThermoFisher Scientific, Waltham, MA, USA).
- MiRNA-133a: Total RNA extraction from serum/plasma samples was carried out using the miRNeasy kit (Qiagen, Hilden, Germany, cat. No 217184), with cel-miR-39 (Qiagen, cat. No 219610) spiked in as an internal control to normalize for variations between samples. Reverse transcription was then performed on 2 ng/μL of RNA using the TaqMan MicroRNA Reverse Transcription Kit (ABI, Carslbard, CA, USA). The resulting cDNA was subsequently analyzed using quantitative PCR (ABI 7500 Fast) with a TaqMan microRNA assay kit to detect miR-133a, following the manufacturer’s protocol. The ΔCT of miR-133a was obtained after normalization to control as ΔCT = mean CT miR-133a—mean CT cel-miR-39, and expressed as 2^−ΔCT^. Fold changes of miR-133a after the procedure were expressed as 2^−ΔΔCT^. Both measurements were conducted by medical biologist researchers who were unaware of the patients’ clinical data.
- NT-proBNP: This is commonly measured using electrochemiluminescence immunoassay (ECLIA) systems on automated analyzers namely Roche Elecsys^®^ (Basel, Switzerland). All laboratory results were verified by a clinical pathology specialist.

### 2.5. Cardiac Rehabilitation Program

The phase II CR program lasted for three months. It comprises an initial assessment session, EKG telemetry sessions for safety during exercise testing, two to three structured exercise sessions per week, and a final 6-Minute Walk Test assessment. Patients underwent exercise testing, physical and laboratory examinations at the beginning and end of the program to evaluate changes [27].

The structured exercise provided is the tailored BEST (Breathing, Endurance, and Strengthening) Exercise Protocol in a structure-based rehabilitation facility. The protocol comprised three core components: breathing exercises, endurance exercise, and strength training exercises as effectively described in prior studies [27,28]. Breathing exercises involved 10 min of deep breathing in a corrective thoracal posture combined with upper-extremity range of motion exercise to optimize chest expansion. Endurance exercise began with a 10 min warm-up focusing on general flexibility for the upper and lower extremities, followed by 20 min of core exercise, such as brisk walking or treadmill use, at a moderate intensity (40–59% Heart Rate Reserve), with intensity subjectively maintained using a talk test to ensure conversational ability [29]. Patients performed breathing and endurance exercises 3–5 days per week. Strength training exercises were conducted on alternating days, targeting major upper-body muscles (pectorals, deltoids, biceps, triceps, and forearm) using lightweight dumbbells (1–2 kg) for three sets of 10–12 repetitions, or major core and lower-body muscles (gluteus, quadriceps, hamstring, and latissimus dorsi) using calisthenics and chair-based exercises. Strengthening sessions were performed 1–2 days per week on alternating days for each major muscle group, in line with ESC guidelines [30].

### 2.6. Statistical Analysis

Data underwent manual review, editing, coding, cleaning, and calculation using IBM SPSS for Macintosh version 20. Numerical data were presented as mean ± standard deviation for normal distributions or median with range for non-normal distributions. Categorical data were presented as counts and percentages. Normality and homogeneity of variance tests were performed. The primary outcome variables were the pre- and post-rehabilitation levels of miRNA-133a and myostatin. Inferential statistics included paired *t*-tests for normally distributed paired data and Wilcoxon tests for non-normally distributed paired data to compare parameters before and after rehabilitation. Chi-square or Fisher’s exact tests were used for categorical data. Pearson correlation was used for normally distributed numerical data, and Spearman correlation for non-normally distributed data. Given the exploratory nature of the correlation analyses and the number of comparisons, there is an increased risk of Type I errors. While *p*-values are reported, adjustments for multiple comparisons were not applied to all analyses; therefore, these correlations should be interpreted with caution and confirmed in larger studies. For non-normally distributed data, such as NT-proBNP, non-parametric tests were employed, as transformations were not considered necessary given the appropriateness of non-parametric approaches.

## 3. Results

### 3.1. Study Population Characteristics

A final cohort of 69 CHF patients who completed the 3-month phase II CR program was randomly allocated and included for biomarker analysis. Within this cohort, the median age was 56 years (range 29–65 years). The study population primarily consisted of males, and the principal cause of heart failure was coronary artery disease. The mean body mass index (BMI) for the total cohort was 26.44 ± 4.90 kg/m^2^, indicating obesity class 1. The median left ventricular ejection fraction (LVEF) for the total cohort was around 29%.

### 3.2. Changes in Physical Parameters and Biomarkers After Rehabilitation

Evaluation of the 69 subjects before and after the 3-month phase II CR program showed significant improvements in several physical parameters (Table 1). Specifically, the 6MWT distance significantly increased from 394.58 ± 62.70 m before rehabilitation to 461.20 ± 74.63 m after rehabilitation (*p* < 0.001). Superior chest expansion increased significantly from 2.03 ± 0.94 cm to 2.81 ± 0.78 cm (*p* < 0.001). Inferior chest expansion also showed significant improvement, increasing from 2.73 ± 1.42 cm to 3.69 ± 1.69 cm (*p* < 0.001). Furthermore, inspiratory diaphragm thickness significantly increased from 0.40 ± 0.16 cm to 0.47 ± 0.14 cm (*p* < 0.001). While median Short Physical Performance Battery (SPPB) score increased from 11 to 12, this change was not statistically significant in the analysis (*p* = 0.268). Dominant side handgrip dynamometry also showed significant improvement (30.60 ± 8.15 kg to 32.37 ± 8.04 kg, *p* = 0.003), as did non-dominant side handgrip dynamometry (28.42 ± 8.08 kg to 29.83 ± 9.13 kg, *p* = 0.030). While median Short Physical Performance Battery (SPPB) score increased from 11 to 12, this change was not statistically significant (*p* = 0.268).

In contrast, the analysis of myostatin and miRNA-133a levels did not show statistically significant changes after undergoing rehabilitation in the subjects (*p* > 0.05 for both). Similarly, anterior forearm muscle thickness on both the dominant and non-dominant sides did not show significant changes after rehabilitation in this group. The median NT-proBNP levels also did not change significantly (*p* = 0.353).

### 3.3. Relationship Between Biomarkers and Physical Examination Parameters

Despite the lack of significant changes in biomarker levels post-rehabilitation, cross-sectional analyses revealed interesting relationships between biomarker levels and physical function parameters at single time points.

**Figure 1:**
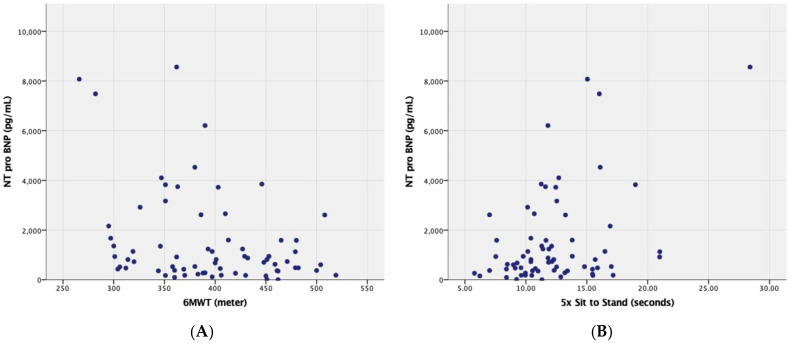
Scatter plots demonstrating cross-sectional baseline correlations between NT-proBNP levels and sensitive physical parameters. (**A**) Inverse correlation between baseline NT-proBNP and 6-Minute Walk Test (6MWT) distance (r = −0.324, *p* = 0.007). (**B**) Moderate correlation between baseline NT-proBNP and 5 times sit-to-stand time (r = 0.413, *p* < 0.001).

- Myostatin: Comparisons of myostatin levels (Table 2) did not reveal statistically significant differences across various physical examination parameters except for diaphragm thickness. There was a trend towards lower myostatin values in subjects with better physical capacity. Lower myostatin levels were found in the group without diaphragm atrophy (defined as expiratory diaphragm thickness ≥ 2 mm, median 687.13 ± 246.03 vs. 829.90 ± 296.45 pg/mL for atrophy, *p* = 0.036). Conversely, and notably, higher mean myostatin levels were observed in patients with better 6MWT performance (mean 856.70 ± 283.23 pg/mL for 6MWT ≥ 400 m vs. 691.56 ± 262.18 pg/mL for 6MWT < 400 m, *p* = 0.014). This contradictory observation, where a biomarker typically associated with muscle wasting is higher in a group with better endurance, warrants careful interpretation in the discussion.
- MiRNA-133a: Mean comparison analysis of miRNA-133a levels (Table 3) across various physical parameters did not show statistically significant differences. However, Pearson correlation analysis (Table 4) revealed significant correlations between miRNA-133a levels and several physical examination parameters. A negative correlation was found with Sit-to-Stand test time (r = −0.282, *p* = 0.019). Positive correlations were observed with 6MWT distance (r = 0.256, *p* = 0.033), superior chest expansion (r = 0.345, *p* = 0.004), and inferior chest expansion (r = 0.337, *p* = 0.005).
- NT-proBNP: Median comparison of NT-proBNP levels showed a reduction trend after 3-month rehabilitation completion (Table 1). This was however not seen to be statistically significant, but similar to the other analyzed biomarkers. NT-proBNP seemed to have weak inverse correlation in a cross-sectional analysis with 6MWT and handgrip strength, while there were moderate correlations for 4 m gait speed and 5 times sit-to-stand (Table 5). To visually illustrate these cross-sectional baseline relationships, scatterplots comparing NT-proBNP levels with 6MWT distance and 5 times sit-to-stand time are provided (Figure 1).

Overall, while these biomarkers of interest (myostatin and miRNA-133a) did not show significant improvement after the 3-month rehabilitation program, significant cross-sectional findings demonstrated that lower myostatin and higher miRNA-133a levels correlated with better overall endurance across cardiovascular, respiratory, and musculoskeletal systems.

## 4. Discussion

### 4.1. Principal Findings and Interpretation

It could be seen how HF significantly impacts not only cardiac function but also extracardiac systems, most notably skeletal muscle. Our study provides further evidence that a 3-month phase II CR program leads to significant improvements in several key physical parameters in CHF patients, including 6MWT distance, chest expansion, inspiratory diaphragm thickness, and handgrip strength. These findings underscore the importance of CR in improving functional capacity in this population.

### 4.2. Comparison with Previous Studies on Biomarker and Novelty in This Study

Despite these observable improvements in physical function, our study did not find significant changes in the circulating levels of myostatin or miRNA-133a after the 3-month CR program. This finding suggests that while CR effectively enhances physical capabilities, its impact on these specific circulating biomarkers might require a longer duration of intervention or different exercise modalities to induce measurable changes. Prior literature on exercise and myostatin/miRNA-133a levels in HF is varied; some studies report changes with longer or more intense interventions, while others suggest these biomarkers are more reflective of chronic disease states rather than acute or short-term exercise adaptations [9,31]. For instance, some research indicates that significant alterations in muscle-related biomarkers may take longer than 3 months to manifest, particularly in conditions of chronic disease.

Nevertheless, the cross-sectional analyses yielded exploratory observations into the relationships between these biomarkers and physical function. For myostatin, we observed lower levels in patients with better expiratory diaphragm thickness (no atrophy), reinforcing its known role as a negative regulator of muscle mass and its association with muscle wasting in CHF. There was also a trend towards lower myostatin levels in patients with normal forearm muscle thickness.

Interestingly, higher mean myostatin levels were observed in patients with better 6MWT performance, a finding that at first glance appears contradictory to myostatin’s established role as an inhibitor of muscle growth [32,33]. However, this observation must be interpreted cautiously. Given that this might reflect differences in muscle fiber type composition or a compensatory adaptive mechanism, our hypotheses are currently speculative and lack supporting data. Furthermore, given that these findings are drawn from subgroup analyses without correction for multiple comparisons, there is a high risk of false-positive correlations. Therefore, these associations should be strictly defined as exploratory.

For miRNA-133a, while grouped comparisons were not significant, its positive correlations with 6MWT distance, superior chest expansion, and inferior chest expansion are noteworthy. This suggests that higher circulating miRNA-133a levels are associated with better endurance and respiratory muscle function in stable CHF patients undergoing CR. The context of exercise training, particularly cardiac rehabilitation in stable CHF patients, might induce a different regulatory response for miRNA-133a in skeletal muscle. It is plausible that in this specific setting, higher miRNA-133a could serve as a protective or adaptive biomarker, actively promoting beneficial skeletal muscle adaptations and inhibiting adverse remodeling in response to physical activity. However, similar to the myostatin findings, the low-level statistical significance drawn from these correlations requires cautious interpretation.

Our study also observed the expected inverse associations between NT-proBNP and 6MWT distance and handgrip strength, confirming its established role as a marker of cardiac dysfunction that inversely relates to physical capacity in HF patients [16,34,35]. The lack of significant longitudinal reduction in NT-proBNP after 3 months of CR, despite improvements in physical parameters, suggests a dissociation between physical fitness and central cardiac markers. Similar to observations in respiratory rehabilitation, where physical fitness often improves in the absence of parallel changes in forced expiratory volume (FEV), the beneficial impact of short-term CR in our HF cohort (presented with a severely reduced median baseline LVEF of 29%) appears to be predominantly peripheral [36]. The program effectively drives skeletal muscle adaptations, but it may not be sufficient or long enough to reverse the underlying central hemodynamic stress and myocardial dysfunction that dictates NT-proBNP secretion and LVEF.

### 4.3. Limitations of the Study

The limitations of our study are important for a balanced interpretation of the findings. Firstly, concerning the study design, the use of a prospective single-group design, without a non-rehabilitated control group, is a major limitation that precludes a definitive attribution of the observed physical improvements solely to the cardiac rehabilitation program. While we observed significant improvements in physical parameters, these changes could be partially influenced by the natural history of the disease or other confounding variables not accounted for in this study. This design limits our ability to make causal inferences about the intervention’s effects.

Secondly, the modest sample size of 69 patients is a notable limitation that affects the statistical power of our analysis. The relatively small sample size and the absence of a formal power calculation weaken the robustness of our findings, limiting our ability to determine true effect sizes or detect subtle longitudinal changes in circulating biomarkers. The small size also restricts the generalizability of our cross-sectional findings and the power to perform more granular subgroup analyses. While all ultrasound measurements were performed by a single highly experienced musculoskeletal radiologist to ensure consistency, formal intra-observer variability testing was not conducted within this specific study cohort.

Furthermore, important confounders such as renal function (eGFR), specific medications alongside their precise dosages, and detailed dietary intake were not controlled in this study. The failure to control for these variables significantly limits the biological interpretation of our results, as these unmeasured factors can independently influence biomarker fluctuations and physical outcomes. Lastly, the study did not delve into the specific muscle fiber type composition or detailed metabolic profiles, which could further elucidate the complex interplay between myostatin, miRNA-133a, and exercise adaptations.

### 4.4. Future Directions

The findings of this study suggest several avenues for future research. Conducting studies with a longer CR duration (more than three months) is recommended to potentially observe more substantial changes in muscle structure and biomarker levels. Future investigations should expand the panel of biomarkers to include a wider range reflecting both myocardial health (such as Galectin-3) and extracardiac function to provide a more comprehensive understanding of the molecular changes in HF patients undergoing rehabilitation. Further research is needed to clarify the underlying mechanisms linking specific extracardiac physical impairments and biomarker levels, which could inform the development of targeted therapeutic interventions, potentially targeting biomarkers like Myostatin to manage sarcopenia. Validating the HARKIT-HF prediction model in larger and more diverse cohorts, potentially including heart failure with preserved ejection fraction (HFpEF) patients, is crucial for establishing its clinical utility. Continued research into myokines and microRNAs is needed to deepen the fundamental understanding of their roles in HF-related skeletal myopathy and explore their potential as biomarkers for personalizing exercise prescriptions.

## 5. Conclusions

While a 3-month cardiac rehabilitation program significantly improves physical parameters in heart failure patients, it did not lead to significant changes in circulating myostatin or miRNA-133a levels during this short-term intervention. However, exploratory cross-sectional analyses revealed trends suggesting that lower myostatin levels and higher miRNA-133a levels may be associated with better physical endurance and function. These findings highlight the potential of these biomarkers as relevant indicators for assessing skeletal myopathy in the HF population, and suggest that practical physical assessments can serve as accessible proxy measures reflecting the underlying myopathic status, even in the absence of detectable longitudinal biomarker changes from short-term CR.

## Figures and Tables

**Table 1:** Baseline Data and Changes after 3-month Phase II Cardiac Rehabilitation.

Extracardiac Parameters	Baseline (n = 69)	After CR (n = 69)	*p*
Age (years)	56 (29–65)		
Female (%)	8 (11.59%)		
BMI (kg/m^2^)	26.44 ± 4.90	25.88 ± 4.21	
SPPB (max 12)	11 (7–12)	12 (9–11)	0.268 ^a^
6-Minute Walk Distance (m)	394.58 ± 62.70	461.20 ± 74.63	<0.001 ^b^
Superior Chest Expansion (cm)	2.03 ± 0.94	2.81 ± 0.78	<0.001 ^b^
Inferior Chest Expansion (cm)	2.73 ± 1.42	3.69 ± 1.69	<0.001 ^b^
Inspiratory Diaphragm Thickness (cm)	0.39 ± 0.14	0.47 ± 0.16	<0.001 ^b^
Dominant Side Handgrip dynamometry (kg)	30.60 ± 8.15	32.37 ± 8.04	0.003 ^b^
Non-dominant Side Handgrip dynamometry (kg)	28.42 ± 8.08	29.83 ± 9.13	0.030 ^b^
Dominant Anterior Forearm Thickness (cm)	2.23 ± 0.65	2.14 ± 0.55	0.291 ^b^
Non-Dominant Anterior Forearm Thickness (cm)	2.09 ± 0.60	2.35 ± 2.10	0.335 ^b^
NTproBNP	1680.01 (85–14,334)	1520.00 (65–8563)	0.353 ^a^
miRNA-133a	30.99 ± 2.31	31.27 ± 2.05	0.475 ^b^
Myostatin (pg/mL)	784.72 ± 257.33	765.76 ± 282.21	0.521 ^b^

All values are presented in Mean ±SD or Median (min-max). ^a^ Analyzed with Wilcoxon Test. ^b^ Analyzed with unpaired *t*-test.

**Table 2:** Myostatin Differences Across Physical Parameter Groups.

Physical Examination	Group	n	Mean	±SD	*p*-Value
6MWT Distance ≥ 400 m	<400 m	38	691.56	±262.18	0.014 ^a^
	≥400 m	31	856.70	±283.23	
Dominant handgrip dynamometry < 30 kg	<30 kg	34	694.87	±253.66	0.110 ^a^
	≥30 kg	35	834.62	±294.83	
Non-dominant Handgrip dynamometry < 30 kg	<30 kg	39	732.10	±289.04	0.262 ^a^
	≥30 kg	30	809.51	±271.61	
Dominant Side Anterior Forearm Ultrasonographic Thickness < 19 mm	≥19 mm	25	691.97	±196.24	0.065 ^a^
	<19 mm	44	807.68	±315.41	
Non-Dominant Side Anterior Forearm Ultrasonographic Thickness < 19 mm	≥19 mm	27	764.68	±230.92	0.979 ^a^
	<19 mm	42	766.45	±313.48	
Inspiratory Diaphragmatic Thickness < 4 mm	Diaphragmatic Dysfunction	33	784.68	±248.92	0.979 ^a^
	Normal (>4 mm)	36	784.75	±268.34	
Expiratory Diaphragmatic Thickness < 2 mm	Diaphragm Atrophy	38	829.90	±296.45	0.036 ^a^
	Normal (≥2 mm)	31	687.13	±246.03	

^a^ Analyzed with unpaired *t*-test.

**Table 3:** MiRNA-133a Differences Across Physical Parameter Groups.

Physical Examination	Group	n	Mean	±SD	*p*-Value
6MWT Distance ≥ 400 m	<400 m	38	30.53	±2.22	0.064 ^a^
	≥400 m	31	31.56	±2.33	
Dominant handgrip dynamometry < 30 kg	<30 kg	34	30.60	±2.33	0.163 ^a^
	≥30 kg	35	31.38	±2.26	
Non-dominant Handgrip dynamometry < 30 kg	<30 kg	39	30.98	±2.14	0.959 ^a^
	≥30 kg	30	31.01	±2.56	
Dominant Side Anterior Forearm Ultrasonographic Thickness < 19 mm	≥19 mm	25	30.82	±2.66	0.640 ^a^
	<19 mm	44	31.09	±2.12	
Non-Dominant Side Anterior Forearm Ultrasonographic Thickness < 19 mm	≥19 mm	27	31.24	±2.79	0.239 ^a^
	<19 mm	42	30.83	±1.97	
Inspiratory Diaphragmatic Thickness < 4 mm	Diaphragmatic Dysfunction	33	30.76	±2.65	0.424 ^a^
	Normal (>4 mm)	36	31.21	±1.97	
Expiratory Diaphragmatic Thickness < 2 mm	Diaphragm Atrophy	38	30.81	±2.48	0.483 ^a^
	Normal (≥2 mm)	31	31.21	±2.11	

^a^ Analyzed with unpaired *t*-test.

**Table 4:** Myostatin Differences Across Physical Parameter Groups.

Physical Examination Parameters	Correlation Coefficient (n = 69)	*p*-Value
6MWT Distance (m)	0.256	0.033 ^a^
4 Meter Gait Speed (m/s)	−0.116	0.342 ^a^
5 Times Sit to stand time (s)	−0.282	0.019 ^a^
Superior Chest Expansion (cm)	0.345	0.004 ^a^
Inferior Chest Expansion (cm)	0.337	0.005 ^a^
Dominant Side Handgrip Strength (kg)	0.227	0.061 ^a^
Non-dominant Side Handgrip Strength (kg)	0.216	0.075 ^a^

^a^ Analyzed with Pearson Correlation Test.

**Table 5:** Correlation NT-proBNP with Various Physical Examination Parameters.

Physical Examination Parameters	Correlation Coefficient (n = 69)	*p*-Value
6MWT Distance (m)	−0.324	0.007 ^a^
4 Meter Gait Speed (m/s)	0.306	0.011 ^a^
5 Times Sit to stand time (s)	0.413	<0.001 ^a^
Superior Chest Expansion (cm)	−0.119	0.329 ^a^
Inferior Chest Expansion (cm)	−0.009	0.944 ^a^
Dominant Side Handgrip Strength (kg)	−0.297	0.013 ^a^
Non-dominant Side Handgrip Strength (kg)	−0.241	0.046 ^a^

^a^ Analyzed with Spearman Correlation Test.

## Data Availability

Data is currently inaccessible to public, however requests could be made in private through the corresponding author e-mail.

## Abbreviations

The following abbreviations are used in this manuscript:

6MWT	6-Minute Walk Test
BEST	Breathing, Endurance, and Strengthening
BMI	Body Mass Index
cDNA	Complementary DNA
CHF	Chronic Heart Failure
CR	Cardiac Rehabilitation
CT	Cycle Threshold
ECLIA	Electrochemiluminescence immunoassay
EDTA	Ethylenediaminetetraacetic acid
eGFR	Estimated Glomerular Filtration Rate
EKG	Electrocardiogram
ELISA	Enzyme-Linked Immunosorbent Assay
ESC	European Society of Cardiology
HF	Heart Failure
HFpEF	Heart failure with preserved ejection fraction
ICC	Intra-class correlation coefficients
LVEF	Left Ventricular Ejection Fraction
miRNAs	MicroRNAs
NT-proBNP	N-terminal pro-B-type natriuretic peptide
PCR	Polymerase Chain Reaction
PJNHK	National Cardiovascular Center Harapan Kita
RNA	Ribonucleic acid
SPPB	Short Physical Performance Battery
TGF-β	Transforming growth factor-beta
USG	Ultrasonography
